# Supplementary material for: Cellular composition and circuit organization of the locus coeruleus of adult mice
Source: eLife. 2023 Feb 3;12:e80100. doi: 10.7554/eLife.80100 (PMC9934863; doi:10.7554/eLife.80100)
Supplement: Figure 2—source data 1. [file elife-80100-fig2-data1.docx]

**Table 1: Electrophysiological Properties of MPs and FFs**

| Cell Type | **MP (n=41)** | | **FF (n=19)** | |
| --- | --- | --- | --- | --- |
| AP threshold (mV) | -31.2 ± 0.9 |  | -30.9 ± 1.5 |  |
| AP amplitude (mV) | 69.6 ± 1.6 |  | 77.4 ± 1.6** |  |
| AP half-width (ms) | 2.20 ± 0.07 |  | 1.84 ± 0.06** |  |
| AP upstroke-to-downstroke ratio | 3.29 ± 0.09 |  | 3.68 ± 0.17* |  |
| Resting membrane potential (mV) | -44.8 ±1.5 |  | -44.9 ± 2.1 |  |
| Input resistance (MOhm) | 710.3 ± 60.0 |  | 707.5 ± 52.9 |  |
| Time constant (ms) | 69.0 ± 5.4 |  | 75.4 ± 5.9 |  |
| AHP (mV) | 23.1 ± 0.8 |  | 25.1 ± 1.6 |  |

***p* < 0.01, **p* < 0.05
